# Cancer prevention at the microscopic level with the potent power of micronutrients

**DOI:** 10.1016/j.heliyon.2024.e39680

**Published:** 2024-10-23

**Authors:** Israt Jahan, Md Aminul Islam, Mohammad Harun-Ur-Rashid, Gazi Nurun Nahar Sultana

**Affiliations:** a Genetic Engineering and Biotechnology Research Laboratory (GEBRL), Centre for Advanced Research in Sciences (CARS), University of Dhaka, Dhaka, 1000, Bangladesh; b Department of Chemistry, International University of Business Agriculture and Technology (IUBAT), Dhaka, 1230, Bangladesh

**Keywords:** Micronutrients and cancer prevention, Antioxidative nutrients, Nutritional oncology, Cellular mechanisms in cancer, Micronutrient clinical trials, Public health and nutrition

## Abstract

Cancer remains a leading cause of morbidity and mortality worldwide, necessitating ongoing exploration of effective prevention strategies. Micronutrients, vital for maintaining cellular health, offer promising avenues for cancer prevention. This review delineates the critical roles of micronutrients in cancer prevention, elucidating their mechanisms at the cellular level. Focusing on essential vitamins and minerals like Vitamins A, C, D, E, selenium, and zinc, we explore their profound effects on fundamental cellular processes such as DNA repair, oxidative stress regulation, cellular proliferation, and immune surveillance. These nutrients, characterized by their antioxidative, anti-inflammatory, and immune-enhancing properties, have shown potential in reducing the risk of cancer. The article synthesizes outcomes from a broad spectrum of clinical trials, epidemiological studies, and systematic reviews to evaluate the efficacy of micronutrients in thwarting cancer development. This critical analysis explores significant trials, addresses controversies in nutrient efficacy, and highlights the implications for clinical practice and public health policy. The review underscores the importance of integrating nutritional strategies into comprehensive cancer prevention frameworks and suggests directions for future research to optimize the preventive potentials of micronutrients.

## Highlights

• Explores essential vitamins and minerals like Vitamins A, C, D, E, selenium, and zinc, emphasizing their roles in DNA repair and oxidative stress regulation for cancer prevention.
• Integrates findings from diverse studies to assess the effectiveness of micronutrients in cancer risk reduction.
• Provides mechanistic insights into how micronutrients enhance immune surveillance and inhibit cancerous cellular proliferation.
• Discusses the integration of micronutrient supplementation into public health strategies for cost-effective cancer prevention.
• Suggests future research directions to optimize the preventive potentials of micronutrients, including personalized nutritional strategies.

## Graphical abstract

**Figure:**
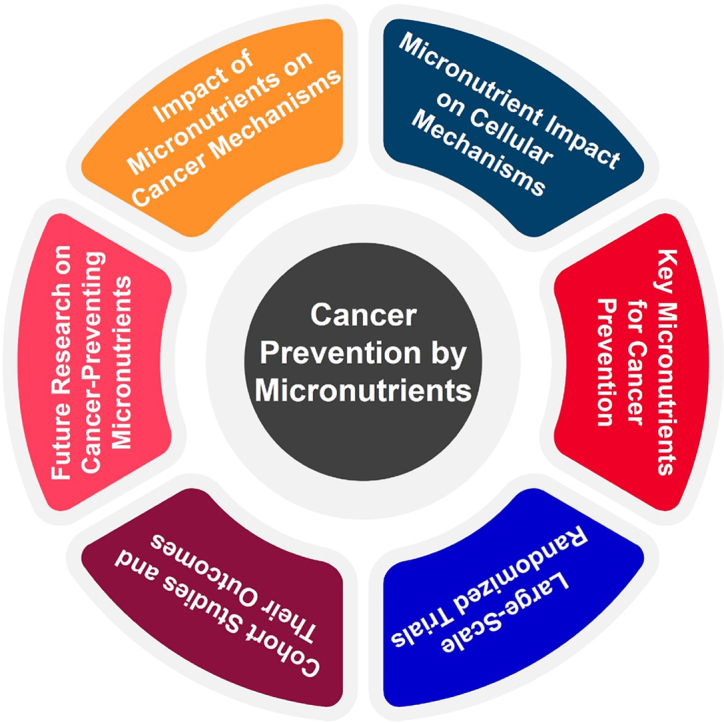

## 1 Introduction

Cancer stands as a formidable global health challenge, claiming millions of lives annually. Despite advancements in medical technology and treatment strategies, the incidence of cancer continues to rise, underscoring an urgent need for effective prevention measures. Among the myriad approaches, the role of micronutrients in cancer prevention offers a compelling avenue for research and application. This review explores the microscopic mechanisms through which micronutrients exert their potent effects to thwart the initiation and progression of cancer, providing a critical synthesis of current knowledge and future directions in this burgeoning field. Micronutrients, including vitamins and minerals, are essential for maintaining normal body functioning and ensuring cellular health. They play critical roles in growth, disease prevention, and overall health maintenance [1]. However, their impact on cancer prevention is particularly significant, given their involvement in key cellular processes such as DNA repair, oxidative stress management, and immune function [2]. These nutrients can influence the very foundations of cancer pathology, offering mechanisms for prevention that are both cost-effective and accessible. Beyond individual nutrient effects, the synergistic impact of multiple micronutrients on cancer prevention is of considerable interest. The interactions between different vitamins and minerals, and their combined effect on cellular health, could potentiate their anticarcinogenic properties [3]. This review examines the synergistic effects of combined micronutrient therapies in cancer prevention, highlighting emerging research and new micronutrients with promising effects on cancer biology. It advocates for integrating nutritional science with oncology to develop more effective prevention strategies. The review aims to inspire further research, influence policy changes, and improve clinical practices, contributing to a new era of preventative strategies that utilize the power of dietary components.

## 2 Rationale and significance

Several previous reviews have explored the role of micronutrients and dietary patterns in cancer prevention, examining the potential benefits and risks associated with specific vitamins, minerals, and overall dietary habits. For instance, Greenwald et al. provided an overview of clinical trials investigating vitamin and mineral supplements for cancer prevention, focusing on large-scale randomized controlled trials like the Linxian Trials, ATBC, and SELECT [4]. This review highlighted mixed outcomes, including potential risks of supplementation, such as increased lung cancer risk among smokers using beta-carotene and vitamin E. Similarly, Vernieri et al. examined the role of dietary patterns in cancer prevention, emphasizing the complex interactions between individual dietary constituents [5]. This review synthesized evidence from systematic reviews and meta-analyses, covering various dietary patterns, such as the Mediterranean diet, and their associations with cancer risk. It offered a broader perspective on how these dietary patterns might influence cancer development. Another significant review by Irimie et al. investigated the relationship between specific dietary factors and cancer risk, focusing on individual nutrients or phytochemicals [6]. This work emphasized the challenges in assessing the influence of single nutrients on cancer risk due to their intercorrelation in typical dietary patterns, adding complexity to the interpretation of their effects. Steck and Murphy further explored the relationship between dietary patterns and cancer risk, analyzing various diets such as the Mediterranean diet and the Healthy Eating Index [7]. This review discussed the latest advancements in dietary patterns research, including the use of metabolomics and statistical techniques to better understand the links between diet and cancer risk. However, these reviews either concentrated on broader aspects of dietary patterns and micronutrient effects or were limited to discussing the epidemiological and clinical trial outcomes. They often lacked a detailed analysis of the underlying cellular and molecular mechanisms by which micronutrients exert their cancer-preventive properties. Additionally, the integration of micronutrient strategies into clinical practice and public health policy was not extensively addressed, leaving a gap in understanding how these findings could be translated into effective cancer prevention strategies.

While previous reviews have provided valuable insights into the role of multivitamin supplementation in cancer prevention and the association between dietary patterns and cancer risk, there remains a significant gap in understanding the detailed cellular mechanisms through which specific micronutrients exert their preventive effects. This review aims to fill this gap by offering a comprehensive synthesis of recent advancements in this area. This article provides an in-depth exploration of how various micronutrients interact with cellular pathways involved in cancer prevention, such as DNA repair, oxidative stress regulation, and immune surveillance. This review also integrates the implications of these findings for both clinical practice and public health policy, advocating for the inclusion of micronutrient strategies in preventive healthcare frameworks. By focusing on the unique cellular mechanisms of micronutrients, the study offers a fresh perspective that not only summarizes current knowledge but also guides future research and policy development. This integrated approach is particularly timely and essential given the increasing burden of cancer globally and the need for cost-effective, accessible preventive strategies.

## 3 Critical evaluation of previous studies on micronutrients and cancer prevention

Previous research on the role of micronutrients in cancer prevention has provided valuable insights, but the findings have been mixed and, in some cases, controversial. For example, large-scale randomized controlled trials (RCTs) like the Linxian Trials and the Alpha-Tocopherol, Beta-Carotene Cancer Prevention (ATBC) Study demonstrated the potential benefits of vitamins and minerals in reducing cancer risk. However, they also highlighted potential risks, such as an increased incidence of lung cancer in smokers supplementing with beta-carotene and vitamin E [4]. This inconsistency underscores the complexity of understanding the impact of individual micronutrients on cancer prevention. In addition, studies focusing on dietary patterns rather than individual micronutrients have provided a broader perspective on the role of diet in cancer prevention. For instance, research examining the Mediterranean diet and other dietary patterns has suggested associations between these diets and reduced cancer risk, emphasizing the potential of a holistic dietary approach [5,7]. However, these studies often do not delve into the specific cellular and molecular mechanisms through which individual micronutrients exert their effects.

Furthermore, some reviews have investigated the impact of specific dietary factors and phytochemicals on cancer risk, shedding light on how individual dietary constituents interact [6]. For example, studies on the roles of polyphenols, flavonoids, and other plant-derived compounds in cancer prevention have highlighted their antioxidative and anti-inflammatory properties. Despite these findings, there remains a gap in understanding the precise molecular pathways these micronutrients influence, particularly in the context of DNA repair, oxidative stress regulation, and immune function. Cuenca-Micó and Aceves conducted a systematic review for exploring the impact of micronutrient intake or supplementation on breast cancer progression, including vitamins (C, D, and E), folic acid, metals, fatty acids, polyphenols, and iodine [8]. In vitro studies indicated antiproliferative, cell-cycle arrest, and antimetastatic effects for most micronutrients. However, these effects were not consistently observed in animal or human studies, with only a few clinical trials showing positive outcomes, particularly with vitamin D and iodine. Limitations included the discrepancy between in vitro and in vivo results, low bioavailability of micronutrients, and tumor heterogeneity. The review emphasized the need for more extensive clinical trials to conclusively establish the antitumor properties of micronutrients in breast cancer progression.

This article addresses the gaps by offering a comprehensive and in-depth analysis of the critical roles that various micronutrients play in cancer prevention. The study examines how specific micronutrients, including vitamins A, C, D, E, selenium, and zinc, interact with cellular mechanisms at a molecular level. By synthesizing outcomes from a broad range of clinical trials, epidemiological studies, and systematic reviews, a balanced perspective on the efficacy and safety of micronutrient supplementation is provided. For instance, the role of selenium in enhancing antioxidant defenses and its impact on various cancers is explored, as well as the intricate balance required in zinc supplementation to modulate immune response without inducing toxicity. Moreover, the controversies and challenges within the field, such as variability in study outcomes and the potential risks of excessive supplementation, are critically evaluated. The importance of a nuanced approach in understanding the role of micronutrients is highlighted, moving beyond simplistic supplementation strategies toward a more integrated framework that considers dietary patterns, individual nutrient interactions, and their combined effects on cellular health. By focusing on the unique contributions of individual micronutrients and their mechanistic roles in cancer prevention, this review builds upon and advances the current body of knowledge. This detailed exploration is crucial for guiding future research, informing clinical practice, and shaping public health policies related to cancer prevention.

## 4 Materials and methods

In conducting this review, we employed a systematic approach to ensure a comprehensive and unbiased evaluation of the available literature. Our methodology included several key components.

### 4.1 Search strategy

We conducted a thorough search using electronic databases including PubMed, Scopus, Web of Science, and Cochrane Library to identify relevant studies up to April 2024. The search terms included combinations and variations of 'micronutrients,' 'vitamins,' 'minerals,' 'cancer prevention,' 'antioxidative properties,' 'DNA repair,' 'immune system,' and 'cellular mechanisms.' Boolean operators were used to refine the search and capture both broad discussions and specific studies related to micronutrients and cancer prevention.

### 4.2 Inclusion and exclusion criteria

Studies were included if they were peer-reviewed empirical research, systematic reviews, or meta-analyses that explored the impact of micronutrients on cancer prevention at the cellular level. Only articles published in English within the last ten years were considered. Studies focusing solely on treatment effects or those that did not provide clear insights into cancer prevention mechanisms were excluded.

### 4.3 Risk of bias assessment

To ensure the reliability of our findings, each study was assessed for potential bias and methodological rigor. We evaluated factors such as study design, sample size, statistical analysis, and clarity in reporting outcomes. Studies were categorized into tiers based on their reliability, with higher-tier studies contributing more significantly to our synthesis of evidence. This qualitative assessment allowed us to consider the weight and validity of the findings in our narrative synthesis.

### 4.4 Data extraction and synthesis

Data extraction was conducted independently by two reviewers using a predefined template, capturing study characteristics, interventions, outcomes, and key findings. Any disagreements between reviewers were resolved through discussion or with the involvement of a third-party adjudicator. We grouped studies thematically by micronutrient type and cancer prevention outcomes, facilitating an aggregated discussion of general trends, consensus areas, and research gaps.

### 4.5 Considerations for systematic review and meta-analysis

While our review aimed to provide a narrative synthesis rather than a quantitative meta-analysis, we acknowledged relevant findings from existing meta-analyses. The heterogeneity in study designs, populations, and outcomes among the included studies precluded the feasibility of conducting a meta-analysis in this review. Nevertheless, we provided a critical evaluation of the current evidence base, discussing the strengths and limitations of the included studies.

By implementing this systematic approach, we aimed to enhance the replicability and transparency of our review, allowing for a comprehensive and critical assessment of the role of micronutrients in cancer prevention.

## 5 Theoretical framework and mechanistic insights

### 5.1 Cellular mechanisms of cancer and micronutrient impact

#### 5.1.1 Genetic mutations

Genetic mutations play a pivotal role in the onset and progression of cancer. These mutations can be inherited or acquired and often result in the disruption of normal cellular functions, including cell cycle regulation, apoptosis, and DNA repair mechanisms. When the DNA repair process is compromised, cells are unable to fix mutations that may lead to cancer, making the integrity of these repair systems crucial for preventing malignancy. Micronutrients have been shown to influence both the occurrence of genetic mutations and the efficacy of DNA repair mechanisms. For example, antioxidants like Vitamin C and E help reduce oxidative stress, a significant contributor to DNA damage that can lead to mutations if not repaired properly [9]. These vitamins can neutralize reactive oxygen species (ROS), thereby reducing the likelihood of mutations and supporting the body's natural DNA repair processes.

#### 5.1.2 DNA repair

10.13039/100014337Furthermore, certain micronutrients play direct roles in supporting 10.13039/100026054DNA repair enzymes. Zinc, for instance, is a cofactor for several proteins involved in the DNA repair process. It helps maintain the structure and function of these proteins, ensuring that they can correctly identify and correct errors in the DNA sequence [10]. Similarly, selenium is involved in the maintenance of endogenous antioxidant systems like glutathione peroxidase, which protect DNA from oxidative damage and help in maintaining genomic stability [11]. Vitamin B complex, particularly folate (vitamin B9), is critical for the synthesis and repair of DNA. Folate deficiency has been linked to increased rates of DNA strand breaks and chromosomal damage, which can elevate cancer risk [12]. Supplementation with folate is considered beneficial in populations at risk of folate deficiency, potentially reducing the risk of cancers associated with DNA damage [13]. Emerging research also suggests that polyphenols, such as those found in green tea and certain fruits, can modulate the activity of DNA repair enzymes and the cellular response to DNA damage [14]. These compounds appear to enhance the body's ability to respond to and repair damaged DNA, providing an additional layer of protection against cancer development. By maintaining genomic stability and enhancing the DNA repair capacity, micronutrients play an essential role in cancer prevention. Their inclusion in the diet or as supplements could be a strategic approach to reducing cancer risk associated with genetic mutations and defective DNA repair mechanisms. The ongoing investigation into the specific mechanisms and interactions of these micronutrients with cellular DNA repair pathways holds significant promise for future preventive strategies in oncology.

Zinc supports DNA repair enzymes, maintaining their function in correcting DNA errors [10], while selenium and Vitamin B complex, especially folate, help protect and repair DNA [11]. Folate, for example, is essential for DNA synthesis and repair, and its supplementation can reduce cancer risks linked to DNA damage [13]. Polyphenols in green tea [15] and certain fruits [16] may boost DNA repair enzyme activity and enhance cellular repair mechanisms. Overall, micronutrients are vital in maintaining genomic stability and preventing cancer by enhancing DNA repair capacities, with ongoing research promising to refine these strategies further.

#### 5.1.3 Oxidative stress

**Fig. 1:**
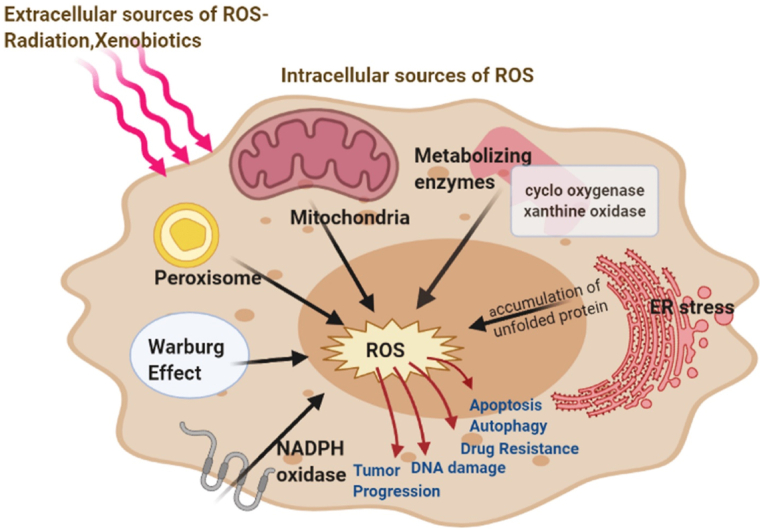
Key internal sources of reactive oxygen species (ROS) include mitochondria, peroxisomes, endoplasmic reticulum (ER) stress, nicotinamide adenine dinucleotide phosphate hydrogen (NADPH) oxidase, and various metabolizing enzymes. External sources such as radiation and xenobiotics also contribute to ROS production. These ROS play a critical role in the initiation and advancement of cancer, impacting the development and progression of the disease. The figure has been reproduced with the permission from Ref. [17]. Copyright 2021 by the authors. Licensee MDPI, Basel, Switzerland.

Oxidative stress, caused by an imbalance in reactive oxygen species (ROS) production and the body's detoxification ability, can lead to cellular damage and carcinogenesis. Fig. 1 illustrates ROS management in the endoplasmic reticulum (ER) [17]. Key enzymes, oxidoreductase Ero1 and NADPH oxidase (NOX), generate ROS through oxidative protein folding, producing hydrogen peroxide (H_2_O_2_) which is neutralized by ER peroxidases like peroxiredoxin 4 and glutathione peroxidases. Excessive ER stress increases ROS, which can be mitigated by reduced glutathione (GSH) to stabilize the redox environment. Increased ROS activates the unfolded protein response (UPR) and PKR-like endoplasmic reticulum kinase (PERK) pathway, balancing cellular stress responses. Additionally, ROS can trigger pathways such as Nrf2, enhancing cellular resilience and lifespan, and may escape through channels impacting further cellular signaling.

#### 5.1.4 Free radicals

Free radicals, produced during oxidative stress, can damage cells and mutate DNA, increasing cancer risk. The body combats these radicals with antioxidants like glutathione, and enzymes such as superoxide dismutase and catalase, but often requires support from dietary micronutrients [18]. Vitamins C and E, along with beta-carotene, are potent antioxidants; Vitamin C reduces free radicals and regenerates other antioxidants, while Vitamin E prevents lipid peroxidation in cell membranes. Additionally, micronutrients like selenium and zinc influence cell growth and apoptosis, supporting cellular defenses and regulating oxidative stress [19,20]. Trace elements such as copper, zinc, and selenium are crucial in the cytosolic defense against reactive oxygen and nitrogen species; they catalyze the conversion of superoxides and reduce peroxides and peroxynitrites, enhancing cellular resistance to oxidative damage [21].

#### 5.1.5 Control of cellular proliferation

Control of cellular proliferation is another crucial aspect influenced by oxidative stress [19,20]. Under normal physiological conditions, cellular proliferation is tightly regulated. However, oxidative stress can alter these regulatory pathways, promoting abnormal cell growth and the potential for malignant transformation. Nutrients like selenium and zinc not only support antioxidant defenses but also play roles in modulating cell growth and apoptosis [22]. Klotz Lars-Oliver and colleagues have reported that the trace elements copper, zinc, and selenium play crucial roles in the cytosolic defense against reactive oxygen and nitrogen species [21]. They note that copper, zinc–superoxide dismutase catalyzes the conversion of superoxide into oxygen and hydrogen peroxide, which are then reduced by the selenoenzyme glutathione peroxidase (GPx). GPx not only acts on hydrogen peroxide but also functions as a peroxynitrite reductase in the cytosol. Beyond their roles in enzyme functions, these trace elements enhance cellular defense mechanisms through other pathways: copper and zinc ions may activate the antiapoptotic phosphoinositide-3-kinase/Akt signaling cascade and stabilize proteins against oxidation. Selenium, present as selenocysteine in GPx and as selenomethionine replacing methionine in proteins, facilitates the reduction of peroxynitrite using glutathione. Furthermore, pharmacologically interesting low-molecular-weight organoselenium and organotellurium compounds have been shown to catalyze the reduction of hydroperoxides or peroxynitrite, utilizing various cellular reducing agents.

Vitamin D also plays a significant role by regulating genes involved in cell cycle and growth, reducing the proliferation of cancer cells in various tissues. Vitamin D, primarily through its active form calcitriol, regulates gene expression by binding to the Vitamin D receptor (VDR), influencing key genes involved in cell cycle control [23]. It increases the expression of cyclin-dependent kinase inhibitors such as p21 and p27, which help regulate cell cycle progression and inhibit proliferation, while reducing the expression of CDK2, a critical protein for cell cycle advancement. Vitamin D also inhibits the Wnt/β-catenin signaling pathway, decreasing the formation of transcription factor 4-β-catenin complexes and enhancing the expression of Wnt antagonist DKK-1, further limiting cancer cell proliferation. These regulatory effects are observed across various cancer types, including breast, prostate, colorectal, and ovarian cancers, demonstrating Vitamin D's significant role in reducing cancer cell proliferation by targeting specific molecular pathways. Research by Chiara Ricca and colleagues also shows that the VDR is essential for maintaining mitochondrial function and preventing oxidative damage, highlighting its critical role in protecting against diseases related to oxidative stress and mitochondrial dysfunction [24].

#### 5.1.6 Inflammatory processes

Inflammation, apoptosis, and cell death are closely related processes vital in both health and disease, including cancer. Chronic inflammation can lead to cellular damage and cancer development. Micronutrients like omega-3 fatty acids, zinc, and selenium can reduce inflammation by inhibiting pro-inflammatory cytokines, potentially lowering cancer risk. Donald C. McMillan and colleagues found that inflammation affects micronutrient levels in gastrointestinal cancer patients, with significant alterations in vitamins and trace elements [25]. Treatment with anti-inflammatory agents showed some improvement in these levels, but they did not fully return to normal, suggesting persistent inflammation. This research highlights the influence of inflammation on micronutrient dynamics in cancer patients and the potential of managing inflammation to modify micronutrient status and improve treatment outcomes. Chronic inflammation's role in diseases like cancer is emphasized by the presence of lymphocytes in tumors and the anti-inflammatory properties of many micronutrients, which also act as antioxidants [26]. Chronic inflammation plays a critical role in cancer development, as it fosters a tumor-promoting environment by contributing to cellular transformation, survival, proliferation, and metastasis. The presence of lymphocytes in tumors indicates an ongoing inflammatory response. Micronutrients, such as vitamins A, C, D, and E, along with minerals like selenium and zinc, exhibit both anti-inflammatory and antioxidant properties, helping to modulate the tumor microenvironment. These micronutrients reduce inflammation by inhibiting key signaling pathways, such as NF-κB and MAPK, while simultaneously neutralizing reactive oxygen species (ROS), preventing cellular damage and supporting immune responses that combat cancer cell proliferation. This dual action of micronutrients on inflammation and oxidative stress makes them potent agents in cancer prevention and management. This dual function is crucial in cancer therapy and nutrigenomics.

#### 5.1.7 Apoptosis

Apoptosis, or programmed cell death, is essential for removing damaged or potentially harmful cells, maintaining cellular homeostasis [27]. Dysregulation of apoptosis is a common feature in cancer, leading to unchecked cell proliferation. Micronutrients like Vitamin E and selenium promote apoptosis in damaged or cancerous cells by affecting key regulators such as Bcl-2 family proteins and caspases. Apoptosis involves a series of events including membrane blebbing, nuclear fragmentation, and chromatin condensation, leading to the formation of apoptotic bodies. The apoptosis pathway is divided into intrinsic and extrinsic pathways, depending on the source of the stimuli. The intrinsic pathway is activated by internal cellular stress, leading to mitochondrial outer membrane permeabilization and the release of proapoptotic factors, which trigger further caspase activation. The extrinsic pathway is triggered by external signals through death receptors on the cell surface, leading to the formation of a signaling complex that activates initiator caspases. These pathways are tightly regulated by the balance between pro- and antiapoptotic proteins, especially the Bcl-2 family, and are influenced by the tumor suppressor p53. Additionally, the extrinsic and intrinsic pathways are interconnected through the action of the Bid protein, which integrates signals from both pathways to amplify apoptotic responses. Understanding these pathways is crucial for developing therapies for apoptosis-resistant cancers and exploring alternative cell death mechanisms like necroptosis, pyroptosis, and ferroptosis, which are increasingly recognized for their roles in cancer.

#### 5.1.8 Cell death

Beyond apoptosis, cell death can occur through mechanisms such as necrosis and autophagy, which also play roles in cancer biology. Necrosis is typically associated with uncontrolled cell death leading to inflammation, whereas autophagy can either promote survival or contribute to cell death, depending on the cellular context and stress level. Vitamins such as Vitamin D and bioactive compounds like polyphenols have been found to regulate these pathways, thus influencing cell survival and death in the context of cancer. Vitamin D, for instance, has been shown to induce autophagy in certain cancer cells, promoting the degradation of damaged cellular components and potentially preventing the progression of cancer [28]. The integration of inflammatory processes, apoptosis, and other forms of cell death through the action of micronutrients illustrates a complex network where nutrient levels and dietary components can dictate cellular fate and cancer risk. Enhancing our understanding of how these micronutrients interact with cellular pathways is key to harnessing their potential in cancer prevention and therapy. Further research is needed to fully elucidate these mechanisms and to determine optimal nutrient intakes for cancer prevention. The roles of micronutrients in modulating inflammatory processes, apoptosis, and cell death are integral to the prevention and potential treatment of cancer. Their impact on these cellular processes highlights the importance of dietary strategies in managing cancer risk and supports ongoing research into nutritional interventions.

### 5.2 Role of micronutrients in modulating cellular mechanisms

#### 5.2.1 Antioxidative properties

Antioxidative properties of micronutrients play a crucial role in combating oxidative stress, which is implicated in various diseases, including cancer. This stress arises from an imbalance between ROS production and the body's ability to neutralize their harmful effects. Antioxidants, such as vitamins C and E and selenium, are vital in reducing ROS overproduction and mitigating cellular damage, especially important in cancer treatment contexts [26]. These micronutrients help neutralize free radicals, protect cell membranes, and maintain cellular redox integrity, thereby preventing DNA damage and carcinogenesis. Antioxidants also modulate key cellular signaling pathways involved in survival, proliferation, and angiogenesis, which are essential for cancer progression. By reducing oxidative stress, they lower the risk of mutations and tumor growth [29]. Furthermore, these nutrients can enhance the body's antioxidant defense system by upregulating endogenous enzymes and regenerating other antioxidants, forming a robust network of cellular protection [30]. Maintaining adequate levels of these antioxidants through diet or supplementation is crucial, particularly for those at risk of or undergoing cancer treatment. Future research is needed to determine optimal micronutrient levels for disease prevention and to understand the complex interactions between dietary antioxidants and body chemistry.

#### 5.2.2 Anti-inflammatory and immune enhancement effects

Micronutrients are essential in modulating inflammatory processes and boosting immune function, crucial for preventing chronic diseases like cancer. Omega-3 fatty acids, vitamins C and D, and minerals like magnesium and zinc significantly reduce inflammation by inhibiting inflammatory cytokines such as TNF-α and IL-6, which are linked to cancer [31]. Vitamin D specifically modulates immune responses, decreasing pro-inflammatory cytokines and boosting anti-inflammatory ones [32]. Additionally, vitamins A, C, E, selenium, and zinc are fundamental to immune health. They support lymphocyte functions—key in combating infections and cancer [33]. Vitamins A, C, E, selenium, and zinc are critical in maintaining immune function by enhancing lymphocyte responses, promoting the production and activity of T cells, B cells, and Natural Killer (NK) cells, all of which are essential in both infection control and cancer surveillance [34]. Vitamin A supports the integrity of epithelial barriers and enhances the function of lymphocytes and NK cells, contributing to the prevention of skin, lung, and gastrointestinal cancers by improving the body's defense against malignancies. Vitamin A maintains mucosal barriers and regulates immune responses, reducing cancer risk [35]. Vitamin C functions as a potent antioxidant and immune enhancer, reducing oxidative stress in immune cells and improving their ability to combat infections. It is also linked to reduced cancer cell proliferation in cancers such as colorectal and lung cancers through its modulation of the inflammatory response. Vitamin E enhances T-cell-mediated immune responses and has been shown to reduce the risk of certain cancers, such as prostate and breast cancer, by neutralizing free radicals and protecting cell membranes from oxidative damage. Selenium is a key micronutrient that works through antioxidant pathways to enhance immune function by promoting the activity of T cells and NK cells. Its deficiency has been linked to an increased risk of certain cancers, particularly prostate, lung, and liver cancers. Zinc supports the development and function of lymphocytes and is particularly important in reducing inflammation and enhancing immune responses in cancers such as esophageal and colorectal cancers. Zinc enhances natural killer cell and T lymphocyte activity, crucial for a robust immune system and managing inflammation [36].

#### 5.2.3 Enhancement of immune surveillance

Micronutrients are vital in enhancing immune surveillance, crucial for detecting and eliminating cancerous cells, thereby aiding in cancer prevention. Vitamins A, C, D, E, and minerals like zinc and selenium bolster immune function. Vitamin D enhances T cell differentiation and NK cell cytotoxicity [37], while Vitamin A supports mucosal barriers and B cell function [38]. Additionally, these micronutrients' antioxidative properties help reduce oxidative stress, maintaining immune cell integrity. Vitamin E, for example, protects lymphocytes from oxidative damage, preserving their functionality [39]. While promising in cancer prevention and treatment, further research is necessary to optimize micronutrient levels and combinations for effective immune enhancement [40]. This understanding is crucial for developing targeted nutritional strategies to boost immune surveillance in cancer prevention and therapy.

## 6 Key micronutrients and their cancer preventive properties

**Fig. 2:**
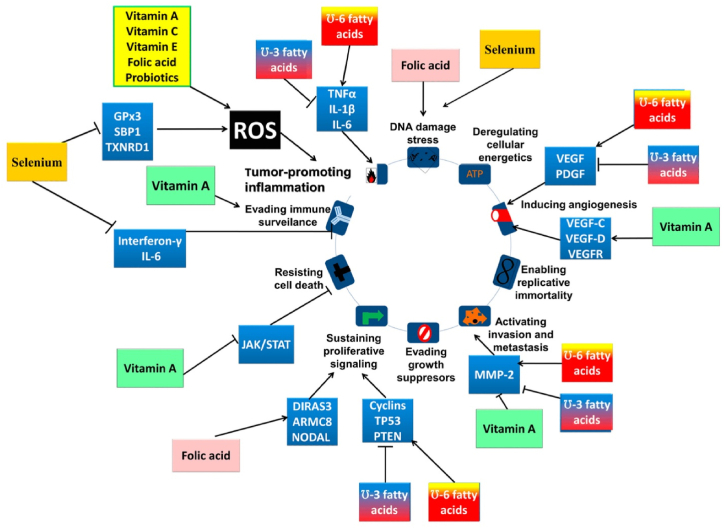
Nutrients impact cancer through their influence on molecular targets associated with cancer hallmarks, primarily by modulating the production of ROS, which play a crucial role in tumor-promoting inflammation. Beyond this, nutrients affect various cancer hallmarks; for instance, fatty acids influence tumor-promoting inflammation, promote angiogenesis, activate invasion and metastasis, and sustain proliferative signaling. These effects significantly alter an individual's susceptibility to cancer and their prognosis. By tailoring dietary interventions, it's possible to develop personalized nutrition strategies that specifically address these aspects, potentially improving cancer outcomes. The figure has been reproduced with the permission from Ref. [6]. Copyright 2019 by the authors. Licensee MDPI, Basel, Switzerland.

Key micronutrients such as vitamins A, C, D, E, and minerals like selenium and zinc are critical in cancer prevention by enhancing immune function, reducing oxidative stress, and modulating gene expression and apoptosis. Vitamin D, for instance, helps prevent the proliferation of malignant cells by inducing cell cycle arrest and apoptosis, while selenium's antioxidant properties protect DNA from damage, lowering mutation and cancer risks [6]. Diets high in processed foods and sugars can increase cancer risk, potentially driving malignant transformations. Although the evidence on the specific anti- or pro-carcinogenic properties of nutrients is mixed, their interaction with molecular pathways related to inflammation and oxidative stress is significant, as shown in Fig. 2.

### 6.1 Vitamins

Vitamin A and its derivatives, carotenoids, are crucial for cancer prevention due to their roles in regulating cell growth, differentiation, and inducing apoptosis, particularly in epithelial tissues. They also act as antioxidants, protecting against oxidative stress and DNA damage, and enhancing immune function [41]. Retinoic acid, a vitamin A metabolite, has been used to treat specific leukemias by promoting cell differentiation [42]. Epidemiological studies show a lower risk of cancers like lung and skin with higher dietary intake of these nutrients [43]. Vitamin C, or ascorbic acid, known for its antioxidant properties and immune system support, plays a significant role in cancer prevention by mitigating oxidative stress and enhancing white blood cell function. It also helps in DNA repair and inhibits angiogenesis, potentially reducing tumor growth and metastasis. High-dose vitamin C has been observed to boost the efficacy of chemotherapy and reduce its toxicity [44]. However, the relationship between vitamin C intake and cancer risk varies, highlighting the need for more controlled trials to establish its cancer-preventive role [45]. Vitamin E, recognized for its antioxidant capabilities, is vital for protecting cell membranes from oxidative damage and modulating gene expression related to cell cycle control and apoptosis. While some studies suggest a protective effect against certain cancers, the evidence is mixed, suggesting that dietary sources rather than supplements might be more beneficial [46]. Vitamin D's role in cancer prevention is linked to its effects on cell differentiation, proliferation, and immune function. Higher levels of vitamin D are associated with a reduced risk of cancers like colorectal, breast, and prostate. However, outcomes from supplementation studies are inconsistent, necessitating further research to clarify its preventive potential [47].

### 6.2 Minerals

Selenium is valued for its antioxidant properties and roles in immune and thyroid function, significantly contributing to cancer prevention by facilitating cellular defense mechanisms and apoptosis in cancer cells. It's integral in selenoproteins that prevent cellular damage [48]. Studies indicate selenium can induce apoptosis in prostate, lung, and colon cancer lines, potentially limiting growth [49]. Epidemiological data suggest an inverse relationship between selenium levels and cancer risk, with mixed results from clinical trials influenced by baseline selenium status and dosage [50]. Zander has potent immune and DNA repair roles, crucial in over 300 enzymatic reactions, stabilizing cell membranes and reducing cancer progression. Zinc deficiency is linked to increased DNA damage and cancer susceptibility, emphasizing its role in apoptosis [51]. Epidemiological studies associate low zinc levels with increased cancer risks, underscoring the need for further investigation [52]. Iron's dual role in cancer involves essential functions like oxygen transport and contributing to oxidative stress, which can increase cancer risks. Iron overload can exacerbate cancer risk, while deficiency may lower colorectal cancer risk. The effects of dietary iron intake on cancer are mixed, requiring more research to determine iron's exact impact [53]. Clinical trials with iron chelators are promising for potentially mitigating cancer risk [54]. Iron levels are best managed through diet, with careful monitoring for those at risk of overload.

## 7 Analyzing evidence

### 7.1 Vitamin studies

#### 7.1.1 Large-scale randomized trials

Analyzing the effectiveness of vitamins in cancer prevention involves diverse research methodologies, including clinical trials, epidemiological studies, and systematic reviews, each offering unique insights into the impacts of vitamin supplementation on cancer risk. Randomized clinical trials (RCTs), viewed as the gold standard, assess the efficacy of vitamins by randomly assigning participants to receive either the supplement or a placebo. This setup allows direct evaluation of the vitamin's impact on cancer incidence and minimizes bias.

Greenwald et al. reviewed several large-scale RCTs and found varying results: trials in Linxian, China, showed a reduction in stomach cancer risk, while studies like the Alpha-Tocopherol, Beta-Carotene Cancer Prevention Study (ATBC) and the β-Carotene and Retinol Efficacy Trial (CARET) reported increased lung cancer risks in high-risk populations [4]. These studies highlight the complex role of nutritional status and the potential interactions among various nutrients affecting cancer risk. The Linxian Trials showed significant cancer mortality reduction in nutritionally deficient populations, while the ATBC trial revealed increased lung cancer risk in smokers taking β-carotene. SELECT focuses on selenium and vitamin E for prostate cancer, but results remain inconclusive. SU.VI.MAX provided moderate evidence for antioxidants in cancer prevention. Limitations include the lack of generalizability for Linxian, harmful effects of β-carotene in smokers (ATBC/CARET), and inconclusive results from SELECT. The WHI trial found no impact of calcium and vitamin D on colorectal cancer, despite prior observational evidence. Conflicting findings arose with β-carotene and colorectal cancer, where observational studies differed from trial outcomes. The trials reviewed, such as the Linxian Trials, Alpha-Tocopherol Beta-Carotene (ATBC) Cancer Prevention Study, and Selenium and Vitamin E Cancer Prevention Trial (SELECT), employed robust study designs, including randomized, double-blind, placebo-controlled methods. These designs help minimize biases and establish clearer links between supplementation and cancer outcomes. Large sample sizes in these studies, often exceeding 20,000 participants, enhance statistical power and generalizability. However, some trials, like the Linxian Dysplasia Trial with 3318 participants, may have limited power for more specific outcomes. Potential biases are also present in these studies. Selection bias, due to the specific populations studied (e.g., smokers in ATBC, nutritionally deficient populations in Linxian), may limit the applicability of the findings to broader populations. Measurement bias, resulting from self-reported supplement use and dietary intake, could introduce recall errors, while varying baseline nutritional statuses and high-dose supplementation may have led to differential effects. Confounding factors, such as lifestyle choices and preexisting conditions, may have impacted results, as seen in the ATBC trial where an unexpected increase in lung cancer risk with beta-carotene was observed among smokers.

Furthermore, Grant and Boucher critiqued the design of vitamin D RCTs, noting that many failed to confirm the protective effects seen in observational studies due to design flaws, such as not considering participants' initial vitamin D levels. They proposed a model to improve RCT designs by accounting for baseline and achieved serum 25-hydroxyvitamin D [25(OH)D] levels, thereby enhancing the reliability of trial outcomes and better understanding vitamin D's role in cancer prevention [55]. These findings underline the need for well-designed clinical trials to clarify the benefits of vitamin and mineral supplementation in cancer prevention, particularly in populations with varying nutritional statuses. The studies show strong observational evidence linking higher vitamin D levels to reduced cancer risk. However, RCTs have yielded mixed results, with some trials like the Nebraska study showing cancer risk reduction only when vitamin D is combined with calcium. Limitations include poor trial design, failure to measure baseline vitamin D levels, and inadequate dosing. Conflicting outcomes emphasize the need for better-designed studies that account for nutrient status and proper dosing to replicate observational findings. The study employs a randomized controlled trial (RCT) design, which is the gold standard for establishing causality. However, the authors argue that many previous vitamin D RCTs were designed using pharmaceutical guidelines rather than nutrient-based guidelines, potentially leading to flawed results. The design of this modeling study addresses these concerns by incorporating Heaney's guidelines, focusing on serum 25(OH)D concentrations rather than just vitamin D dosage. Additionally, the study suggests improvements, such as starting with a bolus dose of vitamin D to achieve the desired concentration and regular monitoring of 25(OH)D levels. The sample size in vitamin D RCTs is a critical factor. The authors model various sample sizes and suggest that achieving statistical significance depends on the baseline 25(OH)D concentrations of participants. For example, the study indicates that 1000 participants per arm are necessary for a baseline concentration of 14 ng/mL, while 4000 participants per arm are needed for a baseline concentration of 26 ng/mL. The reliance on sufficient participant numbers to detect hazard ratio significance is highlighted as crucial for the validity of the results. The study included several potential biases such as participant health, baseline vitamin D status, measurement and compliance, and co-supplementation with calcium.

Gaziano et al. conducted a comprehensive trial with 14,641 male U.S. physicians to assess the impact of long-term multivitamin supplementation on cancer risk, with follow-ups from 1997 to 2011 [56]. Participants were randomized to receive either a daily multivitamin or a placebo. Results showed a modest but significant reduction in total cancer incidence with a hazard ratio of 0.92, although no significant effects were noted on prostate or colorectal cancers. This study suggests a potential benefit of multivitamin use in reducing overall cancer incidence, particularly in individuals with a prior history of cancer. The study's strengths include its large sample size of over 14,000 male participants, its randomized, placebo-controlled design, and a long follow-up period of 11.2 years, which provided valuable insights into cancer incidence and prevention. Additionally, the study found a modest but statistically significant reduction in total cancer incidence among those taking daily multivitamins, especially for cancers other than prostate cancer. However, limitations include the lack of significant findings for individual site-specific cancers like prostate or colorectal cancer, which remained unaffected by multivitamin use. The study also primarily involved well-nourished, male physicians, which limits the generalizability of the results to other populations, including women or those with poor nutritional status. Furthermore, the formulation of the multivitamin changed over time, which could affect the consistency of results. Conflicting results were observed with regard to cancer types. While total cancer incidence was reduced, no significant reduction was seen for prostate or colorectal cancers, contradicting some earlier observational studies. Additionally, although some studies suggest potential cancer prevention benefits from multivitamins, others, such as the Women's Health Initiative, found no association between multivitamin use and cancer reduction. These discrepancies highlight the complexities and need for further research. The Physicians' Health Study II was a large-scale, randomized, double-blind, placebo-controlled trial. This robust design minimizes selection bias and ensures that neither participants nor researchers know who is receiving the multivitamin or placebo, reducing the risk of performance or detection bias. However, the study was focused only on male physicians, which may limit the generalizability of the findings to the broader population, including women and non-physician populations. The study included 14,641 male US physicians aged 50 years or older, providing a large and statistically powerful sample size. The large sample contributes to the study's ability to detect even modest differences in cancer incidence between the multivitamin and placebo groups. However, the inclusion of a highly specific demographic (older male physicians) may introduce sample bias and limit the applicability of the results to other populations. The study included several potential biases such as healthy volunteer bias, adherence and drop-outs and follow-up duration.

Chandler and colleagues explored the role of vitamin D3 (2000 IU/d) in preventing advanced cancer within the VITAL study, involving older adults free from cancer [57]. They found a significant reduction in metastatic or fatal cancer in the vitamin D3 group, especially among participants with a normal body mass index, highlighting the influence of BMI on vitamin D3's effectiveness in cancer prevention. The VITAL trial examined the effects of vitamin D3 supplementation on advanced cancer, showing both strengths and limitations. Strengths include the large, diverse sample size of over 25,000 participants and the randomized, double-blind, placebo-controlled design. The study found a significant reduction in advanced cancers (metastatic or fatal) among those taking vitamin D, particularly in participants with a normal body mass index (BMI). This suggests vitamin D's potential role in reducing advanced cancer risk in individuals with a healthy weight. However, limitations include the lack of effect in individuals with overweight or obesity, indicating a possible interaction between vitamin D efficacy and BMI. Additionally, no significant differences were observed in overall cancer incidence or specific site cancers, except for a modest reduction in prostate cancer mortality. Conflicting results arose from the lack of effect in certain cancer types and BMI categories, emphasizing the need for further research to explore these inconsistencies and refine understanding of vitamin D's role in cancer prevention. The VITAL trial was a randomized, double-blind, placebo-controlled, 2x2 factorial clinical trial, which is a strong design for minimizing biases. Randomization and blinding reduce the likelihood of selection and performance biases. The study specifically evaluated the effects of vitamin D3 and omega-3 fatty acids on cancer prevention, and the factorial design allowed for the investigation of both supplements. However, the study focused only on participants aged 50 years or older who were free of cancer and cardiovascular disease at baseline, which may limit the generalizability to younger populations or those with existing conditions. The study included 25,871 participants (51 % female), which is a large sample size providing sufficient statistical power to detect significant differences. This large cohort is a major strength, especially for detecting relatively rare outcomes such as advanced (metastatic or fatal) cancer. However, the study only followed participants for a median period of 5.3 years, which may not be enough time to observe the full effects of vitamin D3 supplementation on cancer development. Potential biases were healthy user bias and adherence and supplement use. Participants were relatively healthy at baseline (free of cancer and cardiovascular disease), and a significant proportion (43 %) were already taking supplemental vitamin D at allowable doses. This could potentially underestimate the true effect of the study intervention, as those already taking vitamin D might have lower risk of cancer. Although adherence to the trial was high (82 % in the vitamin D group and 80 % in the placebo group), non-study use of vitamin D was higher in the placebo group, which could dilute the observed effects of the intervention. Additionally, there was no control for multiple hypothesis testing in secondary outcomes, which increases the risk of Type I errors.

Manson et al. in the VITamin D and OmegA-3 TriaL (VITAL) examined the effects of daily vitamin D3 (2000 IU) and marine omega-3 fatty acids on cancer and cardiovascular disease prevention among over 25,000 U S. adults aged over 50 [58]. The study found no significant reduction in total invasive cancer incidence or cardiovascular events, although there was a potential reduction in cancer mortality over longer periods, suggesting a delayed benefit from vitamin D supplementation. Yang et al. reviewed the efficacy of different forms of vitamin E in cancer prevention, noting that while α-tocopherol has shown no preventive effect in North American studies, γ- and δ-tocopherols, and tocotrienols may offer stronger preventive activities based on laboratory and animal studies [59]. Their findings suggest potential benefits of these tocopherol and tocotrienol forms in human cancer prevention. Virtanen et al. assessed the effects of vitamin D3 supplementation on cardiovascular and cancer incidence among older adults in Finland over five years [60]. Despite a well-designed trial, no significant effects were observed, likely due to the participants' sufficient baseline vitamin D levels, indicating that supplementation might not benefit populations with adequate vitamin levels. Table 1 distills complex information from the research articles into key points that can help in understanding the broader implications of vitamin supplementation in cancer prevention across different contexts and populations.

**Table 1:** Summary of trends and insights from large-scale randomized trials on vitamin supplementation for cancer prevention.

**Trend/Insight**	**Explanation**
Variable Efficacy of Vitamins	Different vitamins show varying levels of efficacy in cancer prevention. For example, tocotrienols are more promising compared to α-tocopherol, while vitamin D and multivitamins have shown limited effects in well-nourished populations.
Importance of Baseline Status	The baseline nutritional status is crucial in determining the outcome of vitamin supplementation. Nutrient-deficient populations benefit more compared to those with adequate nutritional levels.
Impact of Demographic Variables	Factors like age and BMI influence the effectiveness of vitamin supplementation. For instance, vitamin D3's preventive effect on advanced cancer is more significant in individuals with a normal BMI.
Long-term Effects and Dosage	Longer follow-up periods may be required to detect significant benefits. The study results vary with different dosages and forms of vitamins, highlighting the need for well-considered dosing strategies in trials.
Safety and Adverse Effects	Vitamin supplementation is generally safe with few adverse effects. However, increased risk in specific groups (e.g., smokers with beta-carotene) suggests that safety profiles may vary and require careful consideration.
Complex Interactions	The effectiveness of vitamin supplements can be influenced by complex interactions within the body and with external factors like diet and health status, suggesting a need for personalized approaches to supplementation.

#### 7.1.2 Cohort studies and their outcomes

Cohort studies are valuable for examining the long-term effects of vitamin intake on cancer prevention, following groups over time to assess how varying levels of vitamin consumption impact cancer risk. Park et al. analyzed data from 13 prospective cohort studies involving 676,141 participants, finding modest inverse associations between higher total intakes of vitamins A, C, and E and reduced colon cancer risk, with multivitamins also linked to decreased risk, potentially due to high folate correlation [61]. Vollbracht et al. evaluated the efficacy of intravenous vitamin C in alleviating side effects of cancer treatments in 125 breast cancer patients. They found significant improvements in symptoms like nausea and fatigue with no reported side effects, indicating the safety and potential benefit of vitamin C during cancer therapies [62]. Schöttker et al. conducted a meta-analysis involving 26,018 individuals to explore the relationship between vitamin D levels and mortality. They found that lower vitamin D concentrations were significantly associated with higher all-cause and cardiovascular mortality, and cancer mortality in individuals with a history of cancer, suggesting a role for vitamin D in cancer prognosis [63]. Zhang et al. performed a dose-response meta-analysis of six studies and found that higher serum vitamin D levels were associated with a 47 % reduction in liver cancer risk, indicating a protective effect of vitamin D against liver cancer [64]. Poorolajal et al. analyzed data from 197 cohort studies to investigate lifestyle and dietary factors affecting breast cancer risk. They noted increased risks with cigarette smoking and alcohol, while physical activity and high fruit and vegetable intake decreased risk. Hormone replacement therapy was linked to higher risks, highlighting the potential of lifestyle modifications in breast cancer prevention [65]. These studies underscore the importance of vitamins and lifestyle factors in cancer risk management and the need for further research to clarify these relationships, particularly through randomized controlled trials for long-term outcomes. Table 2 summarizing the key findings from the cohort studies reviewed, focusing on the role of various vitamins in cancer prevention. This table provides a concise overview of the vitamins studied, the types of cancer investigated, and the main outcomes of each study, illustrating the nuanced role of vitamins in potentially mitigating cancer risk across different contexts.

**Table 2:** Summary of cohort studies investigating the role of vitamins in cancer prevention.

**Study (Lead Author)**	**Vitamin Focus**	**Cancer Type**	**Cellular Mechanisms of Cancer Prevention**	**Key Findings**
Park et al. [61]	Vitamins A, C, E	Colorectal Cancer	Antioxidant properties reduce oxidative stress and inflammation	Modest inverse association between intake of vitamins A, C, E, and lower risk of colorectal cancer, particularly in distal colon.
Schöttker et al. [63]	Vitamin D	Various, including mortality rates	Anti-inflammatory, immune modulation, apoptosis	Vitamin D may reduce cancer mortality in individuals with history of cancer.
Zhang et al. [64]	Vitamin E	Liver Cancer	Regulates cell proliferation, differentiation, apoptosis, anti-angiogenesis	Inverse association between high serum vitamin D levels and reduced risk of liver cancer.
Poorolajal et al. [65]	Multivitamin Use	Breast Cancer	Supports DNA repair and reduces oxidative damage	Regular use of multivitamins, including vitamin C and E, associated with reduced risk of breast cancer.
Zschäbitz et al. [66]	Folate, Vitamin B	Colorectal Cancer	DNA methylation and repair, cell proliferation inhibition	Folate supplementation was associated with a lower risk of colorectal cancer, supporting the role of B vitamins in DNA repair.
Peters et al. [67]	Vitamin E	Prostate Cancer	Inhibition of cell proliferation, induction of apoptosis	Vitamin E supplementation showed potential in reducing prostate cancer risk through its antioxidant effects.
Hua et al. [68]	Vitamin C	Pancreatic Cancer	Antioxidant properties, DNA protection from oxidative damage	A meta-analysis showed a significant inverse association between high vitamin C intake and reduced pancreatic cancer risk in case-control studies.
Shibata et al. [69]	Vitamins A, C, E	Bladder, Colon, Lung Cancer (women)	Antioxidant activity, immune function enhancement, inhibition of cell proliferation	Vitamin C supplementation showed reduced risk of bladder cancer in men and colon cancer in women, with protective effects also seen for lung cancer.

Research underscores the intricate link between nutrient intake and cancer prevention, suggesting that specific vitamins, combined with lifestyle factors, could help reduce the risk of certain cancers. These findings support incorporating vitamin considerations into public health guidelines and emphasize the need for more definitive randomized trials to verify these links and clarify causality. Cohort studies, valuable for their long-term insights, face challenges like confounding factors and difficulties in accurately measuring vitamin intake. Future research should focus on refining dietary assessments, using genetic data to identify responsive populations, and exploring how vitamins interact with other dietary and lifestyle elements. This ongoing research is crucial for developing precise, effective cancer prevention strategies that integrate vitamin consumption.

### 7.2 Mineral studies

#### 7.2.1 Selenium trials

Selenium is extensively studied for its cancer-preventive properties, primarily due to its antioxidant capabilities. The Nutritional Prevention of Cancer (NPC) trial highlighted selenium's role in significantly reducing the incidence of cancers such as prostate, lung, and colorectal by enhancing antioxidant defenses and modulating immune functions. Research by Christen et al. in the Selenium and Vitamin E Cancer Prevention Trial (SELECT) examined the effects of selenium and vitamin E on cataract development in over 11,000 men but found no significant benefits of supplementation in reducing the risk of cataracts over a mean follow-up of 5.6 years [70]. Lü et al. analyzed failures in high-profile trials like SELECT to identify shortcomings in the antioxidant hypothesis and the types of selenium compounds used, advocating for the development of next-generation selenium compounds for cancer chemoprevention [71]. Kuria et al. conducted a systematic review and meta-analysis, demonstrating that a daily intake of at least 55 μg of selenium significantly reduced cancer risk, supporting selenium's protective role against cancer [72]. Brodin et al. explored using selenoprotein P (SELENOP) as a biomarker for selenium status in clinical settings, finding that SELENOP levels responded well to high doses of selenium, suggesting its potential for monitoring selenium-based interventions [73]. Ward-Deitrich et al. investigated the effects of different doses of selenium supplementation on human plasma, revealing potential adverse effects on selenoprotein function from high and prolonged selenium doses, which is crucial for designing future trials [74]. Walsh et al. conducted a 6-month randomized, double-blind, placebo-controlled trial to evaluate the effects of selenium supplementation on musculoskeletal health in 120 postmenopausal women with osteopenia [75]. Participants received daily doses of sodium selenite (50 μg or 200 μg) or a placebo. The study focused on changes in urinary biomarkers, bone density, and physical function. Results showed no significant improvement in bone turnover, bone density, or physical performance, concluding that selenium supplementation at these levels did not benefit musculoskeletal health in this group.

Yuan et al. utilized a Mendelian randomization approach to assess selenium's impact on cancer risk across various cancers, finding no broad preventive effect but nothing specific associations with kidney cancer and multiple myeloma, suggesting a complex relationship between selenium levels and cancer risk [76]. Rataan et al. reviewed selenium's role in cancer treatment, focusing on its chemopreventive and therapeutic potentials, particularly in enhancing drug responses and managing drug-resistant tumors [77]. Yang et al. evaluated the effects of selenium supplementation during chemoradiotherapy in cervical cancer patients, noting a significant reduction in hematologic toxicity without affecting overall treatment efficacy or liver and kidney functions [78]. These studies collectively emphasize the nuanced role of selenium in cancer prevention and treatment, advocating for further research to refine supplementation strategies and understand selenium's biological impacts more comprehensively. Table 3 offers a comprehensive overview of various studies related to the impact of selenium on cancer risk and treatment, highlighting the diversity of outcomes and the need for further research in specific areas.

**Table 3:** Overview of the clinical trials on selenium for cancer prevention.

**Authors**	**Study Focus**	**Participant Details**	**Cancer Types Involved**	**Cellular Mechanisms of Cancer Prevention**	**Key Findings**
Christen et al. [70]	Effect of selenium and vitamin E on cataracts and cancer risk	Over 11,000 men from SELECT trial	Prostate cancer (primary focus), incidental findings on other cancers	Selenium's antioxidant role via selenoproteins, reducing oxidative damage, protecting DNA from mutations	No significant reduction in cataract or cancer incidence observed with selenium supplementation in Se-adequate populations
Lü et al. [71]	Review of selenium compounds for chemoprevention	Analysis of previous trials	Prostate, lung, and non-small cell lung cancers	Antioxidant properties of selenium compounds potentially reduce DNA damage and improve immune response, critical in cancer prevention	SeMet and Se-yeast showed no efficacy in Se-adequate groups; next-gen Se compounds recommended for further research in cancer chemoprevention
Kuria et al. [72]	Dietary selenium intake and cancer risk	39 prospective studies, global	General cancer risk, including prostate and lung	Selenium's antioxidant activity supports cell homeostasis, potentially lowering cancer risk by reducing free radicals	Modest protective effect against cancer in men with Se levels within RDA; effects vary by cancer type, suggesting tailored supplementation strategies
Brodin et al. [73]	Selenoprotein P as a biomarker for selenium status	Clinical trials with high-dose Se	Various cancers, emphasis on therapeutic dosage	Selenoprotein P aids in oxidative stress management, potentially lowering mutation rates, essential in cancer prevention	Effective biomarker for selenium status in high-dose therapeutic applications, promising for monitoring selenium treatment
Ward-Deitrich et al. [74]	Selenium fractionation in plasma in cancer prevention	Elderly subjects in UK and Denmark	Prostate, lung, colorectal, bladder	Selenium incorporated into selenoproteins regulates antioxidant enzymes, mitigating DNA damage in specific cancer pathways	Higher Se intake (>100 μg/day) altered selenium distribution in plasma; highlighted need for cautious long-term supplementation
Walsh et al. [75]	Effect of selenium on musculoskeletal health in older women	120 postmenopausal women with osteopenia in the UK	Not cancer-specific, related to oxidative damage	Selenium's antioxidative properties were hypothesized to protect against bone resorption, a process that could impact cancer cell environment indirectly	No significant effects on musculoskeletal health observed, underscoring selenium's limited impact on non-cancer endpoints
Yuan et al. [76]	Selenium levels and cancer risk in Mendelian randomization study	UK Biobank data, 367,561 participants	Kidney cancer, multiple myeloma	Potential inverse relationship with kidney cancer due to selenium's role in reducing oxidative stress and mutation rates	No strong associations found; suggestive inverse correlation with kidney cancer, needing further study for confirmation
Rataan et al. [77]	Therapeutic role of selenium in enhancing drug responses	Preclinical and clinical review with xenograft models and clear-cell renal carcinoma patients	Clear-cell renal-cell carcinoma, head and neck cancer (preclinical)	Selenium modulates drug efficacy by stabilizing vasculature, improving drug delivery, and reducing drug resistance	Higher therapeutic response and prolonged survival in ccRCC patients with selenium combined with axitinib; underscores potential of selenium as a therapy adjunct
Yang et al. [78]	Selenium's effect on chemoradiotherapy for cervical cancer	104 women with stage IIB cervical cancer in China	Cervical cancer	Selenium's antioxidative properties may protect blood cells from treatment-related damage	Selenium supplementation reduced hematologic toxicity and thrombocytopenia in patients receiving chemoradiotherapy without affecting treatment efficacy

Overall, these studies collectively underline the nuanced role of selenium in health and disease, suggesting both potential benefits and limitations. The evidence points to selenium's complex interaction with biological systems, which can vary greatly depending on the form of selenium used, the dosage, the duration of supplementation, and individual patient factors. Further research is needed to delineate these relationships more clearly, particularly in controlled clinical settings, to optimize selenium's potential as a preventive and therapeutic agent in cancer and other diseases.

#### 7.2.2 Zinc supplementation studies

Zinc plays a critical role in cellular metabolism, immune function, and antioxidant systems, influencing cancer risks, especially esophageal and colorectal cancers. However, the impact of zinc supplementation on cancer prevention has been inconsistent, indicating a need for further research to clarify its role. Prasad et al. highlighted zinc's pivotal role in cancer prevention and its deficiency affecting nearly 2 billion globally. Their research showed that 65 % of head and neck cancer patients were zinc-deficient, impacting their immune response and suggesting that zinc supplementation could modulate oxidative stress and inflammation, potentially reducing tumor burden and enhancing treatment outcomes [79]. Figueiredo Ribeiro et al. found that zinc supplementation during chemotherapy for colorectal cancer improved antioxidant defenses, indicated by higher superoxide dismutase activity, although other oxidative stress markers were unaffected. The study underscores the complexity of oxidative dynamics during cancer therapy and the potential protective role of zinc [80]. Hoppe et al. reviewed the effects of zinc on managing cancer treatment-related side effects, particularly radiotherapy-induced mucositis. While beneficial in specific contexts, zinc showed no significant impact on chemotherapy-induced side effects or overall survival, suggesting its benefits are more pronounced in targeted applications rather than broad cancer treatment [81]. These findings suggest zinc's selective benefits in cancer management, particularly for enhancing immune function and managing certain treatment-related toxicities. Further targeted research is required to fully understand and optimize zinc's therapeutic potential in oncology.

## 8 Organized overview of micronutrients and their cancer-specific effects

Micronutrients play vital roles in cancer prevention through mechanisms such as antioxidative properties, modulation of cellular processes, and immune system enhancement. Table 4 provides a structured overview, detailing the specific effects of key vitamins and minerals on various cancer types. It highlights the mechanisms of action, cancer types influenced, and key findings from recent research, offering a concise reference for understanding the multifaceted impact of these essential nutrients in reducing cancer risk.

**Table 4:** Overview of micronutrients and their cancer-specific effects, highlighting the mechanisms of action, impact on various cancer types, and key research findings.

**Micronutrient**	**Cancer Types Addressed**	**Mechanism of Action**	**Specific Impact on Cancer**	**Ref**
Vitamin A	**Lung**	**Antiproliferative Effect**: Vitamin A and its derivatives have antiproliferative effects via growth arrest signaling, promotion of differentiation, and induction of apoptosis. These effects are primarily mediated through retinoid receptors (RAR and RXR) that function as transcription factors modulating gene expression.	**Limited Clinical Evidence**: While preclinical studies have shown positive effects, clinical evidence for Vitamin A in preventing or treating lung cancer is limited. Some RCTs found no significant effect, whereas the CARET trial even found increased risk of lung cancer with the use of Vitamin A and beta-carotene in smokers.	[82]
		**Suppression of Proliferative Markers**: Retinoic acid downregulates markers such as hTERT, cyclins D1 and 3, and growth factors like EGFR and VEGF, inhibiting tumor growth, angiogenesis, and metastasis.	**Event-Free Survival**: In some studies, Vitamin A was associated with a small improvement in event-free survival (RR 1.24), suggesting a possible benefit in reducing progression.	
		**Retinoid Receptors**: Vitamin A exerts its effects through retinoic acid receptors (RAR and RXR), leading to G1 cell cycle arrest and modulation of key signaling pathways involved in cancer progression.	**Synthetic Derivatives**: The synthetic rexinoid bexarotene showed promising results in improving survival in a subset of patients, indicating potential for targeted use of Vitamin A derivatives.	
	**Leukemia**	**Induces Differentiation**: ATRA induces differentiation of promyelocytic leukemia cells by dissociating the histone deacetylase complex from the PML-RARα fusion protein, promoting coactivator binding and transcription.	**Improved Survival**: ATRA, combined with chemotherapy, significantly improved 5-year overall survival (OS: 87 %) and event-free survival (EFS: 76 %) compared to conventional therapy.	[83]
		**Reduces Coagulation Activity**: ATRA downregulates procoagulant activity in promyelocytic blasts, reducing the risk of fatal bleeding events.	**Reduced Early Deaths**: Use of ATRA reduced early deaths from severe bleeding and sepsis, showing significant improvement in patient outcomes.	
		**Enhances Immune Function**: Increases maturation of neutrophils, leading to enhanced immunological function and reduced infection risk.	**Manageable Side Effects**: Common side effects included headaches, fever, and retinoic acid syndrome, which were manageable with dose adjustment and supportive treatment.	
	**Malignant Melanoma**	**Inhibition of Growth and Proliferation**: ATRA binds to retinoic acid receptors (RAR) in cancer cells, inhibiting growth, proliferation, and promoting differentiation.	**Reduced Cell Proliferation**: The combination of ATRA and DBZ significantly reduces melanoma cell proliferation compared to individual treatments. **Increased Apoptosis**: ATRA promotes apoptosis, contributing to the suppression of melanoma cells. **Inhibition of Cell Migration**: The combination treatment notably decreases cell migration, reducing the potential for metastasis.	[84]
		**Synergistic Action with Dacarbazine**: The combination of ATRA and dacarbazine (DBZ) enhances anticancer efficacy through apoptosis induction, cell cycle arrest, and inhibition of migration.		
**Vitamin C**	**Breast Cancer**	**Pro-apoptotic Effect**: High-dose Vitamin C induces apoptosis in breast cancer cells by increasing reactive oxygen species (ROS), leading to DNA damage and cell death.	**Reduced Proliferation**: High-dose Vitamin C significantly reduces the proliferation of breast cancer cell lines (e.g., MDA-MB-231, MCF-7, and SK-BR3) without affecting normal cells. **Enhanced Effect with Anti-cancer Agents**: Combining high-dose Vitamin C with chemotherapy agents (e.g., tamoxifen, eribulin mesylate, trastuzumab) further inhibits breast cancer cell growth compared to using the chemotherapy agent alone. **Effectiveness on Drug-resistant Cells**: High-dose Vitamin C inhibits growth in chemotherapy-resistant breast cancer cells, suggesting a role in overcoming resistance.	[85]
		**Reduced Catalase Activity**: Cancer cells, such as MDA-MB-231 and MCF-7, have lower catalase activity compared to normal cells, allowing ROS accumulation and enhanced pro-oxidant activity of Vitamin C.		
	**Gastric Cancer**	**Antioxidant and Inhibition of Carcinogenic Compounds**: Vitamin C acts as an antioxidant and inhibits the formation of carcinogenic N-nitroso compounds in the stomach.	**Inverse Association with Plasma Vitamin C**: High plasma Vitamin C levels were associated with a reduced risk of gastric cancer (OR = 0.55 for the highest vs. lowest quartile).	[86]
		**Quenching Reactive Oxygen Species (ROS)**: Vitamin C scavenges reactive oxygen species produced in the gastric environment, limiting oxidative damage in gastric epithelial cells.	**Effect Modulation by Diet**: The inverse association between plasma Vitamin C levels and gastric cancer risk was more pronounced in individuals with higher consumption of red and processed meats.	
	**Colorectal Cancer (CRC)**	**Induces Apoptosis**: High-dose Vitamin C induces apoptosis in CRC cells, particularly those with high MALAT1 expression, by increasing reactive oxygen species (ROS) and oxidative stress.	**Suppression of Tumor Growth**: High-dose Vitamin C suppresses CRC growth in both xenograft and peritoneal implantation metastasis models.	[87]
		**Cell Cycle Arrest**: Vitamin C leads to S-phase arrest, reducing the proliferation of cancer cells.	**Inhibition of Metastasis**: High-dose Vitamin C significantly reduces metastasis in mouse models, suggesting its role in limiting cancer spread.	
		**Reduction of MALAT1 Expression**: High-dose Vitamin C reduces MALAT1 expression, which is associated with increased CRC progression, thereby inhibiting tumor growth.	**Enhanced Sensitivity in High MALAT1 Cells**: CRC cells with higher MALAT1 expression are more susceptible to Vitamin C treatment, indicating a targeted effect based on genetic expression profiles.	
	**Colorectal Cancer**	**Selective Uptake via GLUT1**: Vitamin C (in its oxidized form, dehydroascorbate or DHA) is selectively taken up by KRAS and BRAF mutant CRC cells via the GLUT1 transporter.	**Selective Cytotoxicity**: High-dose Vitamin C selectively kills KRAS and BRAF mutant CRC cells by exploiting their glycolytic dependency, resulting in reduced tumor growth in both in vitro and in vivo models.	[88]
		**Oxidative Stress and GAPDH Inhibition**: The uptake of DHA causes oxidative stress, depletes glutathione (GSH), and inactivates glyceraldehyde 3-phosphate dehydrogenase (GAPDH), leading to an energetic crisis and cell death in glycolysis-dependent cancer cells.	**Inhibition of Tumor Growth**: High-dose Vitamin C significantly reduces tumor growth in KRAS and BRAF mutant xenograft models, indicating its potential therapeutic effect against these types of mutations.	
**Vitamin D**	**Colorectal Cancer**	**Cell Differentiation and Immune Modulation**: Vitamin D, in the form of calcitriol, binds to vitamin D receptors (VDR), promoting cellular differentiation and modulating the immune response.	**Reduced Cancer Incidence**: Epidemiological evidence suggests an inverse association between high plasma levels of Vitamin D (25(OH)D) and reduced risk of CRC.	[89]
		**Inhibition of Proliferation and Induction of Apoptosis**: Calcitriol inhibits cancer cell proliferation by inducing G1 phase cell cycle arrest via upregulation of CDK inhibitors (e.g., p21, p27) and promoting apoptosis through upregulation of pro-apoptotic proteins (e.g., BAK1, BAX).	**Tumor Growth Inhibition**: Calcitriol treatment has shown to inhibit tumor growth in colorectal cancer models by targeting multiple pathways, including WNT signaling and CTNNB1 activity.	
		**Anti-Angiogenesis**: Calcitriol inhibits angiogenesis by reducing VEGF expression and endothelial cell proliferation.	**Immune System Modulation**: Vitamin D enhances immune cell function, which is associated with a reduction in CRC risk, particularly in tumors with high immune infiltration.	
	**Breast Cancer**	**Growth Arrest and Apoptosis**: 1,25(OH)2D induces cell cycle arrest by upregulating cyclin-dependent kinase inhibitors (e.g., p21, p27) and promoting apoptosis through downregulation of anti-apoptotic proteins (e.g., Bcl-2, Bcl-XL) and upregulation of pro-apoptotic proteins (e.g., Bax, Bak).	**Reduced Breast Cancer Risk**: Epidemiological studies suggest an inverse association between serum 25(OH)D levels and breast cancer risk, with higher levels correlating with reduced risk.	[90]
		**Inhibition of Invasion and Metastasis**: 1,25(OH)2D increases E-cadherin expression, inhibits matrix metalloproteinases (MMPs), and suppresses angiogenesis, reducing the ability of breast cancer cells to invade and metastasize.	**Lower Cancer Recurrence and Mortality**: Higher levels of Vitamin D are associated with reduced risk of breast cancer recurrence and mortality in women with early-stage breast cancer.	
		**Estrogen Pathway Inhibition**: 1,25(OH)2D suppresses the synthesis and biological action of estrogens by downregulating aromatase enzyme and estrogen receptor (ER) expression, thereby reducing estrogen-driven breast cancer cell proliferation.	**Improved Prognosis**: Sufficient Vitamin D levels (>30 ng/mL) are linked to improved prognosis and survival rates in breast cancer patients.	
	**Prostate Cancer**	**Inhibition of EMT**: Calcitriol (active Vitamin D) inhibits epithelial-mesenchymal transition (EMT) by downregulating EMT-related genes (e.g., Zeb1, Snail) and reducing the interaction between β-catenin and TCF4, which are crucial for prostate cancer cell invasion and metastasis.	**Reduced Growth and Metastasis**: Vitamin D deficiency was found to aggravate prostate cancer growth and metastasis, while calcitriol treatment inhibited tumor growth in two prostate cancer mouse models. **Inhibition of Invasion and Migration**: In prostate cancer cell lines (PC-3 and DU145), calcitriol effectively inhibited cell migration and invasion, contributing to reduced metastatic potential.	[91]
		**Promotion of Adherens Junction Formation**: Calcitriol promotes the formation of the β-catenin/E-cadherin complex, enhancing cell adhesion and maintaining epithelial integrity, which helps suppress tumor spread.		
		**Suppression of β-catenin Signaling**: Calcitriol reduces β-catenin phosphorylation and decreases its transcriptional activity with TCF4, inhibiting proliferation and migration of prostate cancer cells.		
**Vitamin E**	**Liver Cancer**	**Antioxidant Activity**: Vitamin E acts as a potent antioxidant by scavenging reactive oxygen species (ROS) and reducing oxidative stress, which is a major factor in liver carcinogenesis. **Chromosomal and Mitochondrial DNA Protection**: Vitamin E supplementation enhances chromosomal stability and reduces mitochondrial DNA (mtDNA) damage, which are associated with decreased tumor progression. **Suppression of Cell Proliferation and Apoptosis**: Vitamin E reduces hepatocyte proliferation and apoptosis by lowering ROS levels and enhancing chromosomal integrity, preventing preneoplastic lesion formation.	**Reduced Tumor Incidence**: Vitamin E reduced the incidence of liver adenomas by 65 % and prevented the development of carcinomas in a transgenic mouse model overexpressing c-myc and TGFa. **Inhibition of Neoplastic Development**: The dietary supplementation of Vitamin E significantly reduced tumor growth and size, demonstrating its effectiveness in inhibiting both the initiation and progression of liver cancer.	[92]
	**Breast Cancer**	**Drug Resistance Reversal**: Vitamin E conjugate (as part of Chitosan/Vitamin E micelles) enhances the uptake of oxaliplatin and reverses multidrug resistance (MDR) in breast cancer cells.	**Decreased IC50 Values**: The micelles significantly reduced IC50 values in both ER+/PR+/HER2− and TNBC cell lines, indicating enhanced efficacy compared to free oxaliplatin. **Enhanced Apoptosis and DNA Fragmentation**: The micelles induced extensive DNA fragmentation, mitochondrial depolarization, and apoptosis, resulting in effective inhibition of breast cancer cell growth. **Reduced Tumor Growth and Nephrotoxicity**: In vivo studies on 4T1(Luc)-tumor-bearing mice showed significant tumor growth inhibition, prolonged survival, and reduced nephrotoxicity compared to oxaliplatin alone.	[93]
		**Mitochondrial Depolarization**: Induces mitochondrial depolarization, which leads to apoptosis in breast cancer cells.		
		**Reactive Oxygen Species (ROS) Generation**: Increases ROS levels, which contributes to DNA damage and apoptosis.		
		**Cell Cycle Arrest**: Causes G2/M cell cycle arrest, leading to inhibited cancer cell proliferation.		
**Selenium**	**Prostate Cancer**	**Modulation of Antioxidant Pathways**: Selenium has been shown to modulate antioxidant pathways, helping to reduce oxidative stress that can contribute to cancer progression.	**Increased Risk with High-Dose Supplementation**: Men with nonmetastatic prostate cancer who consumed selenium supplementation of 140 μg/day or more after diagnosis had a 2.60-fold higher risk of prostate cancer mortality compared to nonusers.	[94]
		**Influence on Apoptosis and Cellular Proliferation**: Selenium impacts apoptosis and inhibits cellular proliferation, which are crucial in cancer prevention and slowing disease progression. However, very high selenium levels may lead to adverse effects, potentially affecting apoptosis and increasing cancer risk.	**No Significant Effect on Biochemical Recurrence**: Selenium supplementation was not associated with a statistically significant effect on biochemical recurrence of prostate cancer.	
		**Potential U-Shaped Dose-Response**: The study suggests a U-shaped dose-response relationship for selenium, where both deficiency and excess can lead to increased risk, indicating the need for an optimal selenium range for beneficial effects.	**Increased Mortality**: Selenium supplementation at high doses increased prostate cancer-specific mortality, particularly among individuals with already sufficient selenium levels.	
	**Lung Cancer**	**Antioxidant Function**: Selenium acts as an antioxidant, supporting selenoproteins that help mitigate oxidative stress, which can contribute to cancer progression.	**Improved Overall Survival in Stage I**: Higher serum selenium levels (>69 μg/L) at the time of diagnosis were significantly associated with improved overall survival in patients with stage I lung cancer. Patients in the highest tertile of selenium levels had an 80-month survival rate of 79.5 %, compared to 58.1 % in the lowest tertile.	[95]
		**Stimulation of Immune Function**: Selenium enhances the activity of immune cells, including cytotoxic lymphocytes and natural killer cells, which can target cancer cells.	**Reduced Mortality Risk**: Patients with higher selenium levels had a reduced risk of death, with a hazard ratio of 2.73 for those in the lowest selenium tertile compared to the highest, indicating the importance of sufficient selenium levels for prognosis in early-stage lung cancer.	
	**Colorectal Cancer**	**Antioxidant Defense**: Selenium, through its role in selenoproteins like Selenoprotein P (SePP), contributes to reducing oxidative stress, which is a known factor in colorectal cancer development.	**Reduced CRC Risk**: Higher selenium concentrations are inversely associated with colorectal cancer risk. This association was found to be statistically significant for women, with an Incidence Rate Ratio (IRR) of 0.83 (95 % CI: 0.70–0.97) per 25 μg/L increase in selenium levels. **Gender-Specific Findings**: The protective effect of selenium was more evident in women compared to men, suggesting a potential role of sex-specific metabolism and response to selenium.	[96]
		**Modulation of Inflammatory Response**: Selenium is involved in regulating the inflammatory response, which plays a role in colorectal cancer progression.		
		**Role of Selenoprotein P (SePP)**: Higher levels of SePP are associated with a decreased risk of colorectal cancer, especially among women. SePP serves as a key transporter of selenium, supporting cellular protection against oxidative damage.		
**Zinc**	**Esophageal Squamous Cell Carcinoma (ESCC)**	**Inflammatory Modulation**: Zinc deficiency (ZD) induces a distinct inflammatory gene signature in the esophageal mucosa, upregulating pro-inflammatory mediators such as S100a8, S100a9, and Cox-2, contributing to an environment conducive to cancer development.	**Increased Cancer Incidence with Zinc Deficiency**: Prolonged ZD in combination with low doses of the carcinogen N-nitrosomethylbenzylamine (NMBA) led to a 66.7 % incidence of ESCC in rats. Zinc sufficiency or replenishment, however, prevented cancer formation. **Reduction of Dysplasia and Neoplasia with Zinc Replenishment**: Zinc replenishment after carcinogen exposure reversed dysplastic and neoplastic changes, demonstrating zinc's protective role against cancer progression.	[97]
		**Suppression of Inflammatory Signature by Zinc Replenishment**: Zinc replenishment reverses the inflammatory gene signature, reducing the expression of numerous inflammation-related genes (e.g., CXC and CC chemokines, Cox-2) and preventing the progression from dysplasia to neoplasia.		
		**Effect on Cell Proliferation**: Zinc deficiency leads to increased cellular proliferation and hyperplasia, whereas zinc sufficiency helps maintain cellular homeostasis by preventing excessive cell proliferation.		
	**Colorectal Cancer (CRC)**	**Antioxidant Defense Enhancement**: Zinc acts as a cofactor for the antioxidant enzyme superoxide dismutase (SOD), which helps neutralize reactive oxygen species (ROS) during chemotherapy.	**Increased SOD Activity**: Patients who received zinc supplementation showed significantly higher SOD activity during chemotherapy compared to the placebo group, suggesting improved antioxidant defenses.	[80]
		**Modulation of Antioxidant Enzymes**: Zinc supplementation increased SOD activity during chemotherapy cycles, which plays a critical role in converting superoxide radicals to hydrogen peroxide, thus reducing oxidative stress.	**Maintenance of Vitamin E Levels**: Zinc supplementation maintained plasma vitamin E levels during chemotherapy, which was not observed in the placebo group. This suggests a protective role of zinc in reducing oxidative damage during cancer treatment.	
		**Maintenance of Antioxidant Vitamins**: Zinc helped maintain plasma vitamin E concentrations, which is crucial for reducing lipid peroxidation during chemotherapy.	**No Effect on Lipid Peroxidation Markers**: Despite increased SOD activity, zinc supplementation did not significantly affect lipid peroxidation markers (MDA and 8-isoprostane), suggesting that the effect of zinc was primarily on enzymatic antioxidant defense rather than directly reducing lipid oxidation.	

## 9 Future directions, emerging research, and policy implications

Advancements in research are enhancing the role of micronutrients in cancer prevention, with significant implications for public health. Studies in molecular biology and nutrigenomics are tailoring nutrition strategies to genetic profiles, exploring interactions with pharmaceuticals and the microbiome. This research will inform future clinical trials and public health policies, potentially leading to tailored micronutrient recommendations and global supplementation programs. The integration of this knowledge into public health strategies and policymaking aims to optimize the preventive potential of micronutrients in reducing cancer risk.

### 9.1 Research gaps and emerging areas

Research on micronutrients in cancer prevention is progressing, yet there is much to explore about less-studied micronutrients and their interactions within the body. While well-known vitamins and minerals like Vitamin C, D, E, selenium, and zinc have been extensively studied, the anticancer potential of trace elements such as manganese, chromium, and molybdenum needs further investigation. Rodríguez-Tomàs et al. emphasized the complex roles of trace elements (TEs) like zinc and selenium in cancer therapy, highlighting their dual potential as essential nutrients and toxic agents at high levels. Their study calls for precise dosing and further research to integrate TEs into personalized cancer treatment plans [98]. Recent advances have also illuminated how micronutrients impact cellular mechanisms crucial for cancer prevention, such as gene expression, DNA repair, and immune modulation. However, the interactions of micronutrients with traditional cancer therapies and the synergistic effects of combining multiple micronutrients are still poorly understood. Additionally, variability in micronutrient impact across different populations indicates a need for more tailored research [99].

#### 9.1.1 Ongoing research and novel micronutrients

Recent studies are exploring the role of manganese in cancer prevention, particularly focusing on its impact in patients with prostate cancer. Manganese plays a critical role in the antioxidant defense system by being a key component of enzymes like superoxide dismutase (SOD), which mitigates oxidative stress—a major factor in cancer development [100]. In a study comparing serum trace elements in prostate cancer patients, it was found that manganese levels were significantly lower in cancer patients compared to healthy controls. This decrease in manganese may impair the body's ability to neutralize oxidative stress, suggesting a link between manganese deficiency and increased cancer risk. This study highlights the importance of manganese as a lesser-known micronutrient with potential cancer-preventive properties, paving the way for further research on its role in cancer mitigation.

Hou et al. explored the role of manganese-based nano-activators in cancer immunotherapy, focusing on its capacity to enhance the body's innate immunity by stimulating the cGAS/STING pathway [101]. This pathway is critical for detecting cytosolic DNA and activating type I interferons, which promote an immune response against tumor cells. Manganese was shown to increase the activation of this pathway, thereby improving the efficacy of immunotherapy. Their study developed manganese phosphate nanoparticles loaded with doxorubicin, which induced DNA damage in tumor cells and released manganese ions, augmenting STING activity. This dual-action approach resulted in increased recruitment of immune cells, such as cytotoxic T lymphocytes and natural killer cells, to the tumor site, enhancing anti-tumor efficacy. The study demonstrated that manganese not only plays a role in cancer prevention through oxidative stress reduction but also boosts cancer immunotherapy by activating immune pathways. This study exemplifies how novel micronutrients like manganese are being researched for their antioxidant properties and their role in enhancing critical immune responses, potentially reducing cancer risk and improving treatment outcomes.

#### 9.1.2 Personalized nutrition strategies and genetic factors affecting Micronutrient efficacy

Recent advances in nutrigenomics are shaping the future of personalized nutrition strategies, where genetic profiles are used to tailor nutrient intake for optimal health outcomes, including cancer prevention. Genetic variations, particularly single nucleotide polymorphisms (SNPs), can affect how individuals metabolize micronutrients such as vitamins D, A, and C. For instance, variations in the Vitamin D Receptor (VDR) gene can influence how effectively vitamin D regulates cellular processes, including apoptosis and immune response, both crucial in cancer prevention.

Malcomson and Mathers in the past highlighted how recent advances in nutrigenomics are paving the way for personalized nutrition strategies, especially in the context of cancer prevention [102]. Their research emphasized that individual genetic variations significantly impact how nutrients are metabolized and utilized in the body, affecting overall health outcomes. For example, polymorphisms in genes related to antioxidant enzymes such as superoxide dismutase (SOD2) have been linked to varying responses to dietary antioxidants like vitamin C and E, influencing cancer risk. Furthermore, they explained that understanding these genetic factors could allow for tailored nutritional interventions, where individuals at higher risk for cancer due to specific genetic variants could receive customized dietary recommendations. This personalized approach is seen as a more effective method to prevent cancer and other non-communicable diseases, moving away from the "one-size-fits-all" model of nutrition towards more precise, individualized strategies based on genomic data. This example underscores how personalized nutrition, informed by an individual's genetic makeup, can enhance the effectiveness of cancer prevention efforts by addressing the unique interactions between diet, genetics, and disease risk.

Emerging studies highlight the interaction between genetics and micronutrient efficacy, suggesting that individuals with specific genetic profiles may benefit more from targeted supplementation. For example, individuals with polymorphisms in genes related to folate metabolism might require higher levels of folate to maintain proper DNA repair mechanisms. Additionally, research into zinc transporter genes indicates that genetic variations can alter zinc absorption and its role in immune function, further underscoring the need for personalized approaches. Personalized nutrition strategies are not only focused on prevention but are also being integrated into therapeutic contexts. Ongoing clinical trials are exploring the role of personalized supplementation, particularly in high-risk populations with genetic predispositions to cancer. Future research will likely expand on the integration of genomics with nutrition, optimizing cancer prevention strategies through individualized dietary interventions that account for genetic variability.

Table 5, a forward-looking table listing emerging micronutrients not yet widely studied for cancer prevention, along with their hypothesized mechanisms and proposed research approaches. This table is intended to guide future studies and funding priorities by highlighting underexplored areas that may offer new insights into cancer prevention. Table 5 provides a structured overview of less commonly studied micronutrients, suggesting potential mechanisms through which they may influence cancer prevention and outlining initial steps for further research in these areas.

**Table 5:** Potential micronutrients for future cancer prevention research.

Micronutrient	Hypothesized Mechanisms of Action	Proposed Research Approaches
Manganese	Antioxidant properties, involvement in enzyme systems	Conduct cohort studies to evaluate manganese's impact on specific types of cancer, explore interactions with other trace elements
Chromium	Enhancement of insulin sensitivity, affects lipid metabolism	Investigate potential roles in metabolic syndromes associated with increased cancer risk, use controlled trials to assess effects on cancer cell growth
Molybdenum	Crucial for several key enzymatic processes, including sulfite detoxification	Study the impact of molybdenum on liver and lung cancers, examine deficiency correlations in cancer populations
Boron	Role in cell membrane function and enzyme activity	Explore anti-cancer properties through animal models, evaluate dietary boron sources and cancer incidence relationships
Nickel	Involvement in DNA synthesis and cellular metabolism	Research cellular responses to nickel supplementation in pre-cancerous conditions, assess nickel's effect on gene expression in cancer cells
Silicon	Connective tissue strength, potential immune modulation	Conduct observational studies to link dietary silicon intake with lower cancer rates, particularly in digestive system cancers

### 9.2 Policy and educational implications

#### 9.2.1 Public health policies and clinical practice guidelines

Public health policies and clinical guidelines play a crucial role in cancer prevention, advocating for diets rich in essential micronutrients based on robust evidence from clinical studies and trials [103]. Current guidelines emphasize the importance of maintaining optimal levels of micronutrients such as Vitamin D, selenium, and folate to prevent cancers like colorectal and breast cancer [104,105]. To ensure the guidelines are aligned with the latest scientific developments, systematic reviews and expert panels should be used to assess the efficacy of these micronutrients and their mechanisms at the cellular level. Furthermore, recommendations should consider individual variability in micronutrient absorption and metabolism, which can affect the response to supplementation. This could be addressed by proposing personalized nutrition strategies, where genetic screening helps to tailor micronutrient intake based on individual genetic profiles and predispositions [106,107]. Policymakers should also focus on improving health equity by targeting interventions for at-risk populations that are more likely to experience micronutrient deficiencies, such as low-income groups or individuals with limited access to nutrient-rich foods. Future updates to these guidelines could integrate findings from ongoing research, allowing for more dynamic and adaptive recommendations that are personalized based on genetic, environmental, and lifestyle factors. This approach would not only help in preventing nutrient deficiencies but also in avoiding excess intake, ensuring a balanced and effective prevention strategy for cancer [108].

#### 9.2.2 Integrating nutrition and oncology

Integrating nutrition into oncology is crucial for comprehensive cancer care, emphasizing the role of diet and micronutrients in affecting cancer outcomes. Preventive nutrition advocates for diets rich in fruits, vegetables, and whole grains, and low in processed foods and red meats, which help reduce cancer risk [7]. During cancer treatment, maintaining nutrition is vital, as malnutrition can impair treatment efficacy and survival. Oncology dietitians provide tailored dietary counseling to manage treatment side effects and prevent malnutrition [109]. Post-treatment, proper nutrition helps prevent cancer recurrence and improve survival, with lifestyle interventions including diet and physical activity recommended for cancer survivors [110]. Emerging research explores how diets and specific micronutrients affect tumor behavior and treatment responses, such as the impacts of dietary patterns on the microbiome and the potential of diets like the ketogenic diet and polyphenols to influence cancer cells and therapy outcomes [111]. Integrating nutrition into oncology involves multidisciplinary collaboration to ensure nutritional strategies are part of the overall cancer care plan, necessitating training for healthcare providers and updating clinical guidelines with the latest research [112]. As research advances, translating findings into practical oncology strategies, supported by robust health policies, is essential for enhancing cancer prevention, treatment, and survivorship.

#### 9.2.3 Addressing nutrient availability and dietary habits

While public health policies emphasize the importance of micronutrient-rich diets in cancer prevention, variability in nutrient availability remains a significant challenge across different regions and populations. For example, access to essential nutrients like vitamin D, selenium, and folate may be limited due to factors such as geographic location, socioeconomic disparities, and agricultural practices. Policymakers should consider regional fortification programs and supplements to address these gaps, ensuring that at-risk populations receive adequate nutrient intake.

In addition, differences in dietary habits, shaped by cultural, environmental, and economic factors, impact how individuals consume and absorb nutrients. Populations with plant-based diets may be deficient in nutrients such as vitamin B12 and iron, while those consuming processed foods may have imbalances in essential antioxidants and minerals. Tailored nutrition education and interventions should be developed, considering these dietary habits, to make the recommendations more applicable and achievable in diverse populations. Efforts to promote sustainable and locally sourced nutrient-dense foods could also alleviate some of these barriers.

Lastly, public health initiatives should promote personalized nutrition strategies, considering individual genetic differences and dietary habits, to optimize micronutrient absorption and efficacy in cancer prevention. Genetic screening may help identify populations that are more vulnerable to nutrient deficiencies or require specific dietary interventions, ensuring that cancer prevention strategies are effective and equitable across various demographic groups.

## 10 Concluding Remarks

The manuscript emphasizes the crucial role of micronutrients like vitamins A, C, D, E, selenium, and zinc in cancer prevention, highlighting their effects on DNA repair, oxidative stress management, and immune modulation. Evidence from clinical trials, epidemiological studies, and systematic reviews suggests a positive but complex link between micronutrient intake and reduced cancer risk. For example, selenium helps maintain cellular integrity, preventing mutations that could lead to cancer. The findings advocate for incorporating these nutrients into cancer prevention strategies through diet and supplementation, encouraging public health policies to emphasize a nutrient-rich diet. The article envisions future research exploring the mechanisms of micronutrients in cancer prevention, including their interactions with genetic factors and drug efficacy. It highlights the potential of personalized nutrition tailored to individual genetic profiles and health conditions to improve prevention strategies. The study calls for a multidisciplinary approach combining nutritional science, oncology, and public health to develop strategies that leverage micronutrients effectively against cancer. This ongoing research aims to deepen understanding of micronutrients' roles, supporting the scientific community in combating cancer more effectively.

## CRediT authorship contribution statement

**Israt Jahan:** Writing – review & editing, Writing – original draft, Methodology, Investigation, Formal analysis, Data curation, Conceptualization, Visualization. **Md Aminul Islam:** Methodology, Data curation, Conceptualization. **Mohammad Harun-Ur-Rashid:** Writing – review & editing, Writing – original draft, Visualization, Software, Resources, Methodology, Investigation, Formal analysis, Data curation, Conceptualization. **Gazi Nurun Nahar Sultana:** Conceptualization, Methodology, Supervision.

## Declaration of competing interest

None of the authors have any conflicts of interest to declare. This includes any financial, personal, or other relationships with other people or organizations within three years of beginning the submitted work that could inappropriately influence, or be perceived to influence, our work.
